# Pseudorabies virus causes splenic injury via inducing oxidative stress and apoptosis related factors in mice

**DOI:** 10.1038/s41598-023-50431-7

**Published:** 2023-12-27

**Authors:** Wei Sun, Shanshan Liu, Yi Yan, Qingyan Wang, Yu Fan, Samuel Kumi Okyere

**Affiliations:** 1 College of Agriculture, Tongren Polytechnic College, Bijiang District, Tongren City, 554300 Guizhou China; 2 National and Local Engineering Research Centre for Separation and Purification Ethnic Chinese Veterinary Herbs, Tongren City, 554300 Guizhou China; 3 College of Animal Science, Wenzhou Vocational College of Science & Technology, Wenzhou, 325006 People’s Republic of China; 4 Department of Pharmaceutical Sciences, School of Medicine, Wayne State University, Detroit, USA

**Keywords:** Antimicrobial responses, Infectious diseases, Pathogens, Virology

## Abstract

Pseudorabies virus (PRV) is an immunosuppressive virus that causes significant damage to the pig industry. This study aimed to investigate the effects of PRV on oxidative stress and apoptotic related in the spleen of mice to provide basis knowledge for further research on the pathogenesis of PRV in mice model. 36 mice were randomly two groups, the control group which only received 200 μL PBS and infection group which was subcutaneously infected with 200 μL of 1 × 10^3^ TCID_50_/100 μL PRV, respectively. Spleen tissues in each group were collected for further experiments at 48, 72, and 96 h post-infection (hpi). Pathological observation was performed by hematoxylin and eosin Y staining. Biochemical and Flow cytometry methods were used to determine the reactive oxygen species profile and apoptosis of the spleen post-infection and apoptosis detection. In addition, q-PCR and Western blot were adopted to measure the apoptotic conditions of the spleen infected with PRV. The results indicated that the reactive oxygen species (ROS) level in the PRV infection group was remarkedly increased (*p* < 0.01) at a time-dependent pattern. Furthermore, the Malondialdehyde levels in the spleen of mice in the infection group increased (*p* < 0.01) in a time-dependent mode. However, the activity of Catalase, Superoxide dismutase, and glutathione peroxidase and the content of Glutathione in the infection group were decreased with the control group (*p* < 0.01) at a time-dependent manner. In addition, the ratio of splenocyte apoptosis in the infection group significantly increased (*p* < 0.01) in a time-dependent manner. In conclusion, PRV infection causes apoptosis of the spleen via oxidative stress in mice.

## Introduction

Pseudorabies virus (PRV), a dsDNA genome, belongs to the α members of the herpesvirus subfamily and is known as the causative agent of pseudorabies (PR)^1^. PR is a contagious disease that causes neurological symptoms and reproductive disorders. PR causes pathological changes such as inflammation and necrosis in parenchymal organs such as the brain, liver, spleen, and kidneys, and the degree of damage to these organs varies among different strains^2^. Studies have demonstrated that PRV disrupts the balance of cellular oxidation and antioxidant, along with the imbalance between reactive oxygen species (ROS) and their intermediates, inducing oxidative stress in cells resulting into steatolysis, proteolysis, nucleic acid degradation in cells, triggering to cell apoptosis^3^, inflammatory response^4^ and immune suppression^5^. PRV infects various mammals, including pigs, ruminants, carnivores, and rodents, hence leads to huge losses in the global animal production industry^6^.

Pigs are the only natural reservoir of PRV^2^. Studies have shown that humans may also be potential hosts of PRV at the genetic level, indicating that PRV can break through species barriers and cause infection in humans^3^. In addition, there have been several reports on encephalitis patient being infected by PRV^7,8^. 

During viral infection, the relative expression of virus-related genes and the activation of innate antiviral response systems leads to an increase in ROS and toxic byproducts of energy metabolism. The imbalance between ROS and antioxidants including superoxide dismutase (SOD), catalase (CAT), glutathione (GSH), and glutathione peroxidase (GSH-Px) in the body leads to oxidative stress, cell death, and tissue/organ damage. ROS and the resulting changes in cellular redox status become one of the inducing factors for cell apoptosis.

The spleen as an immune organ is one of the major targets of PRV, however, the molecular mechanisms involved in PRV infection and toxicity of the spleen are not well established in literature. Therefore, in this work, the effects and molecular mechanism of PRV on spleen toxicity in mice was investigated, aiming to give a theoretical basis for the key pathogenesis study of PRV. 

## Materials and methods

### Reagents and animals

Six-week-old SPF BALB/c mice was bought from Da-shuo Experimental Animal Co., Ltd, China. The PRV-HLJ strain (MK080279.1) was provided by the Animal Pathogen and Pathological Morphology Innovation Team of the Harbin Institute of Veterinary Medicine, CAAS, and was preserved in our laboratory. The detail information used in this study was listed in Table 1.

**Table 1:** Detail information for the reagents.

Name	Sources	Product Number	Country
ROS detection kits	Thermo fisher scientific-CN	88-5930-74	China
Annexin V-FITC/PI double staining	BD Company	556547	United States
2 × SYBR Green PCR Mastermix	TAKARA Bio	RR820A	China
Reverse transcription kit	TAKARA company	RR047A	China
Tissue RNA extraction kit	Shanghai Sangong Biological Co., Ltd	B518621	China
T-SOD	Nanjing Jian-cheng Biotechnology Research Institute	A001-1-2	China
MDA		A003-1	China
GSH		A006-2	China
GSH-Px		A005	China
CAT		A007-1	China

### Experimental design

36 mice were randomly divided into 2 groups, the control group (n=18) which was subcutaneously administered with 200 μL of PBS and the infection group (n=18) subcutaneously administered with 200 μL dose of 1×10^3^ TCID_50_/100 μL PRV. This dose was selected based on our previous studies we observed the invasion of PRV in various tissue especially the spleen after subcutaneous injection of 0.2 mL PRV-HLJ strain^9^. Mice were kept in a temperature (22–24) and humidity(40–60%) controlled room with a 12 h light and dark cycle. Water and diet were given adlib. At 48, 72, and 96 h post-infection (hpi), mice in each group were anesthetized in an anesthesia chamber filled with 2.5% sevoflurane of O_2_ at a flow rate of 0.9−1 L/min for up to 6 h and then sacrificed. Their spleen tissues were then collected and stored at − 80 for further experiments.

### Tissue microscopy

The fresh spleen in 2.2 was fixed in 10% formalin. Then, the tissues were dehydrate and embedded in paraffin blocks. The blocks were then cut into 5 μM pieces thick. Typically, 4-5 µM slices were cut and fixed on a glass slide. Paraffin was removed by xylene and alcohol. Observe and photograph the tissue structure of the tissue stained with hematoxylin and eosin Y (H.E) using a digital camera.

### Detection of ROS by flow cytometry in the Spleen

Mix spleen tissue were homogenously mixed with DCFH-DA in a volume ratio of 300:1, and then incubated in the darkroom at 37 °C for 20 min. Next, the treated tissue above was centrifuged at 4 °C for 5 min, and followed by the addition 1 mL PBS solution. The supernatant was discarded and resuspended into 400 μL PBS. ROS was detected by using flow cytometry.

### Detection of ROS-related factors in the Spleen

The spleens were collected from each group and ground to form 10% tissue homogenate through with a tissue homogenizer by using a mechanical method. Then, the homogenate of all tissue was centrifuged at 2500 *rpm* for 10 min at 4 °C. The corresponding reagent kits were used to detect the changes in cellular oxidative stress-related factors including MDA, SOD, GSH, GSH-Px, and CAT.

### Detection of splenic cell apoptosis

Fresh spleen tissues were taken from each experimental group and cut into 1 mm^3^ size. The tissue homogenates were prepared mechanically by grinding and passing through a 300 mesh filter. The cell concentration was adjusted to 1×10^6^/mL following by centrifugation and resuspension. Apoptosis with Annexin-V-FITC double stained kit was applied to measure the influence of PRV on the apoptosis of mouse splenocytes.

### RNA extraction and qRT-PCR analysis

Following by the manufacturer’s instruction, "Animal Total RNA Separation Kit" was used to separate total RNA from the spleen. RNA integrity was determined by 1.5% agarose gel electrophoresis. A PrimeScript RT kit was used to convert total RNA to cDNA for qRT-PCR analysis. Next, cDNA was amplified by a PrimScript RT reagent Kit on the LightCycle 96 device (Roche, Germany) following the protocol provided. All oligonucleotide primers for this study were designed by Primer software and synthesized at Sagon Biotech Ltd, China. All qRT-PCR reactions system were mixed with the SYBR staining. Detailed information on primers can be found in Table 2. All relative expression data was calculated by the 2^−ΔΔCt^ method. And β-actin was introduced as an internal reference gene in this study.

**Table 2:** Primers information for qRT-PCR.

Gene	Primer name	Sequence	Product size (bp)
Caspase-3	Caspase-3 F	5′-GGCTGACTTCCTGTATGCTTACTCTAC-3′	96
	Caspase-3 R	5′-ATGCTGCAAAGGGACTGGATGAAC-3′	
Caspase-9	Caspase-9 F	5′-GTGAAGAACGACCTGACTGCCAAG-3′	103
	Caspase-9 R	5′-GAGAGAGGATGACCACCACAAAGC-3′	
Bax	Bax-F	5′-CGTGAGCGGCTGCTTGTCTG-3′	127
	Bax-R	5′-TGGTGAGCGAGGCGGTGAG-3′	
Bcl-2	Bcl-2 F	5′-CGGGAATGCCTACGAACTCTTCAC-3′	100
	Bcl-2 R	5′-ACGGTAGCCAAAGCCCAGGAG-3′	
β-actin	Actin-F	5′-TCCTGACAACACAACTGGTTCACC-3′	137
	Actin-R	5′-TCCATGTCGTCCCAGTTGGT-3′	

### Western blot analyses

“Tissue Total Protein Extraction Kit” was used to extract of the total protein from spleen in all groups. Then, protein concentrations in each group were measured according to the Bradford method. Protein samples were first separated on 10% SDS-PAGE solution and the transferred onto PVDF membranes. Next, PVDF membranes were cut to the appropriate size and incubated with the corresponding primary antibody at 4 °C for 12 h, i.e Caspase-3 (1:900), Caspase-9 (1:1000), Bax (1:800), Bcl-2 (1:600) and β-actin (1:2000), followed by blocking in 5% skimmed milk solution for 2 h, respectively. The protein blot bands were washed three times with 5% PBST solution and reacted with HRP-labeled Goat anti-rabbit antibody for 1h with slight shaking. The membrane was observed on ECL after washing with PBST. The expression level of the proteins of interest to β-actin was calculated by using ImageJ2x software.

### Statistical analysis

SPSS22.0 software was used to analyse the results, and the experimental data were expressed in the form of mean±standard deviation (mean±SD). One-way analysis of variance (ANOVA) algorithm was applied to evaluate the statistical differences among PRV infection and the control groups. The data histograms were plotted by GraphPad Prism 6.0 software. *p*<0.05 and *p*<0.01 value was introduced to determine the significant of differences.

### Ethical statement

This study was authorized by the Animal Ethics Committee of Tongren Polytechnic College. All animal operation and procedures were conducted according to the approved guidelines and were in accordance with the International Guide for the Care and Use of Laboratory Animals and .ARRIVE’s guidelines.

## Results

### Pathological observation on Spleen

**Figure 1:**
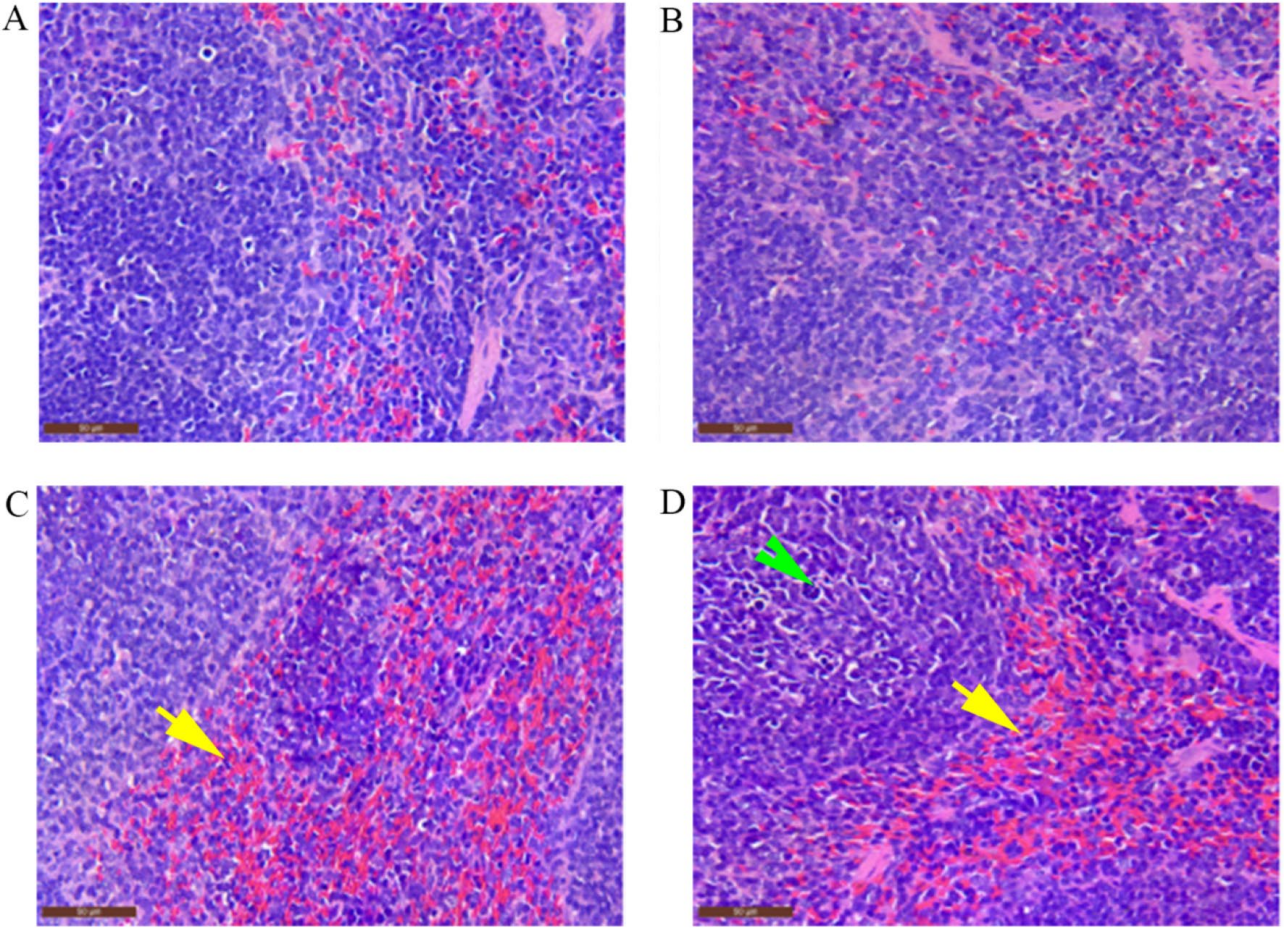
The pathological observation on Spleen (400 × , Bar = 50 μm). (**A**) Control; (**B**) 48 hpi; (**C**) 72 hpi; (**D**) 96 hpi congestion (yellow arrow) and infiltration of neutrophils (green arrow).

Based on the microscopic examination (Fig. 1A and B), the structure of the spleen tissue was normal, and no obvious pathological damage was observed in the control and 48 hpi group. However, we observed different pathological changes in the red pulp area, as well as congestion (Fig. 1C and D, yellow arrow) and focal infiltration of neutrophils (Fig. 1D, green arrow), while no abnormalities were observed in the white pulp area.

### The influence of PRV on ROS levels in the Spleen

**Figure 2:**
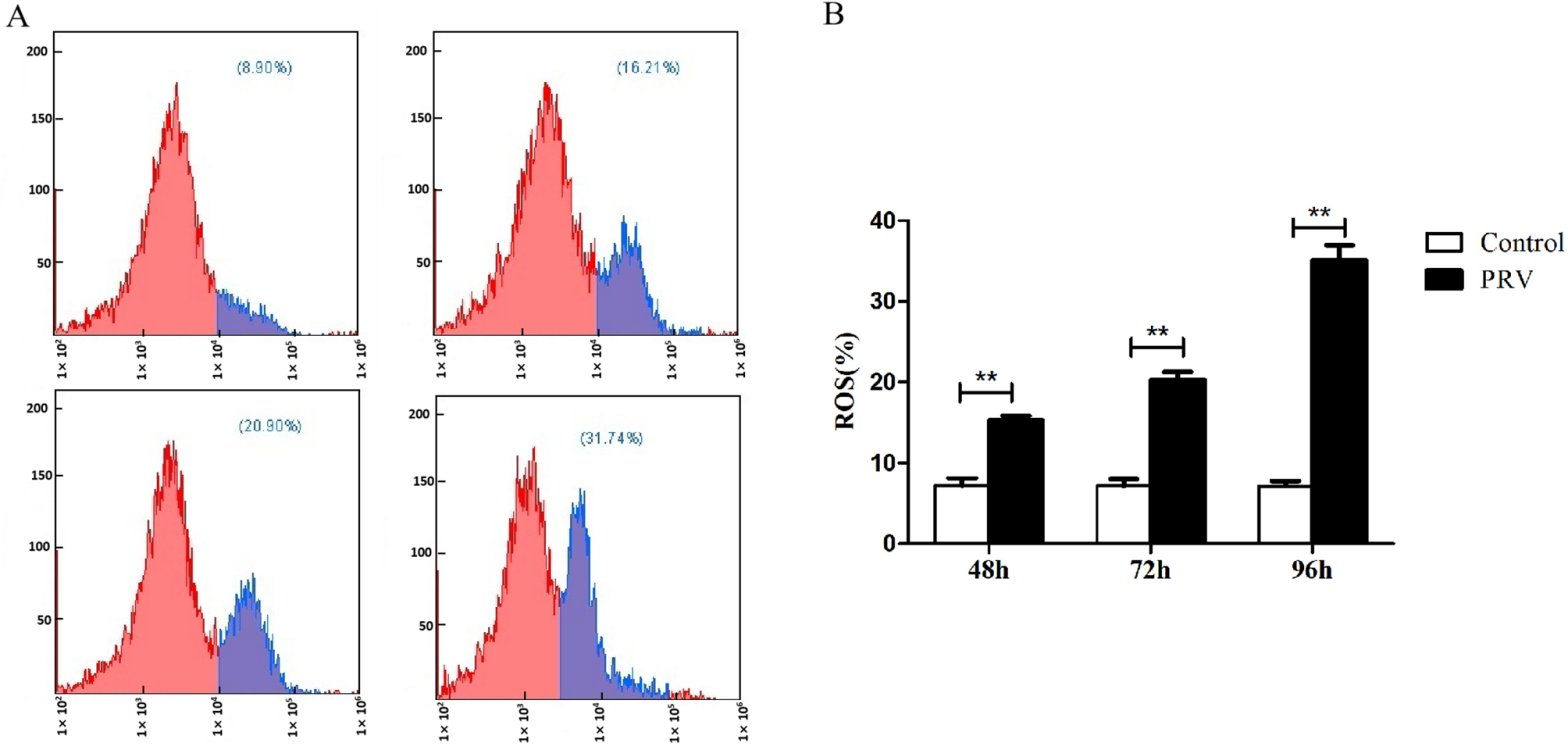
The inducement of PRV on production of ROS in the spleen. (**A**) The results of ROS detected by flow cytometry; (**B**) ROS statistical analysis histogram. **p < 0.01 versus mock infection, the same as below.

The results from the flow cytometry indicated that, compared with that in their respective control groups, ROS production in the spleen of the PRV infection group significantly increased in the whole infection period (*p*<0.01, Fig. 2).

### The effect of PRV on oxidative stress-related factors in the spleen 

**Figure 3:**
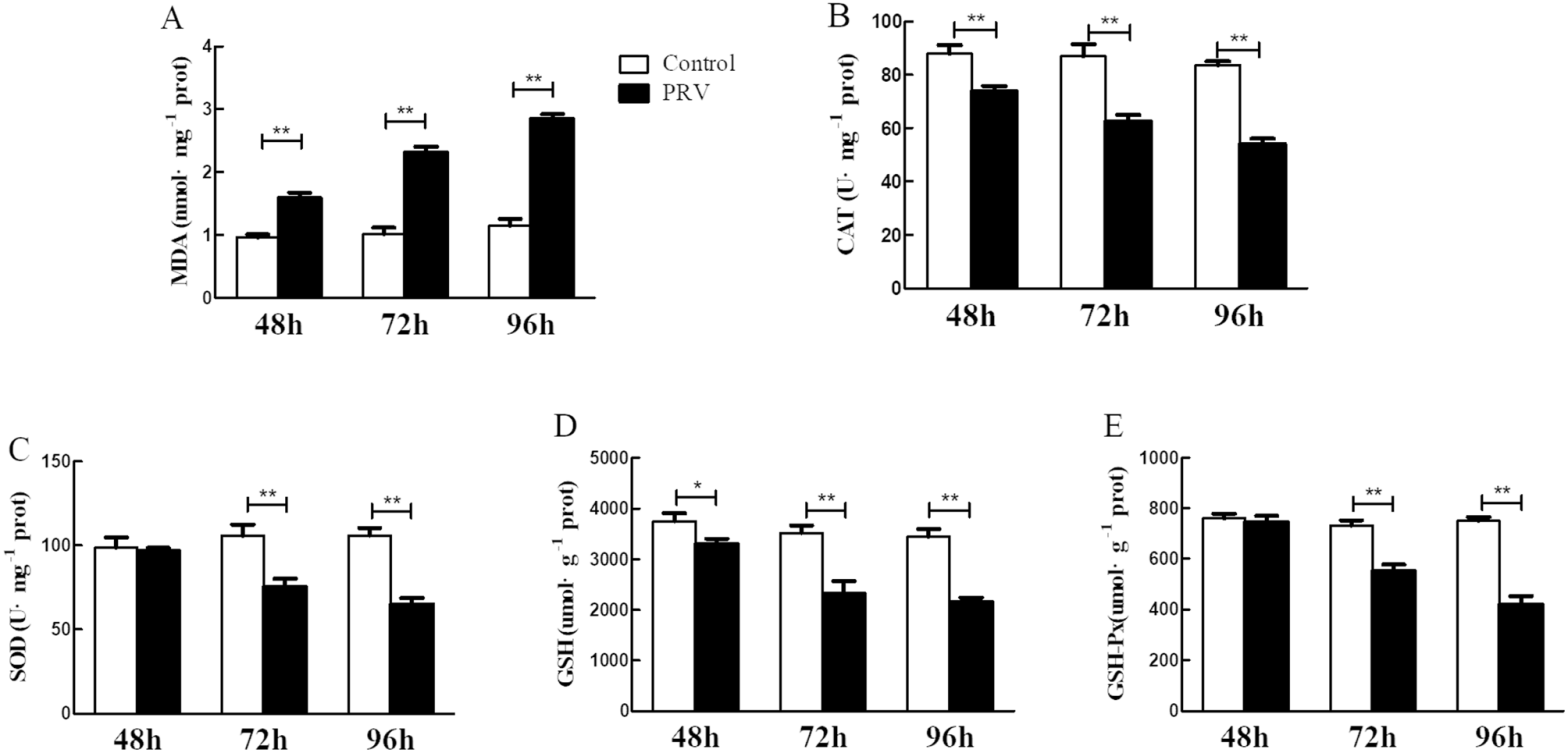
The effect of PRV infection mitochondrial oxidative stress-related factors. (**A**) MDA; (**B**) CAT; (**C**) SOD; (**D**) GSH; (**E**) GSH-Px. *p < *0.05* versus mock infection, the same as below.

As shown in Fig. 3, the PRV treatment group significantly promoted the production of MDA in the spleen more than their control groups (*p *< 0 01) in time dependent manner. However, PRV significantly descends the levels of CAT and GSH in the spleens in a time dependent manner (*p *< 0 05, *p *< 0 01). In addition, compared with the control group, SOD and GSH-Px activities at the time point of 48 hpi showed no statistical difference (*p *> 0.05). However, there was a decrease in both the levels of SOD and GSH-Px in the PRV infection group at the 72 hpi and 96 hpi time points compared to their respective controls (*p *< 0.01).

### Detection of the splenocytes apoptosis

**Figure 4:**
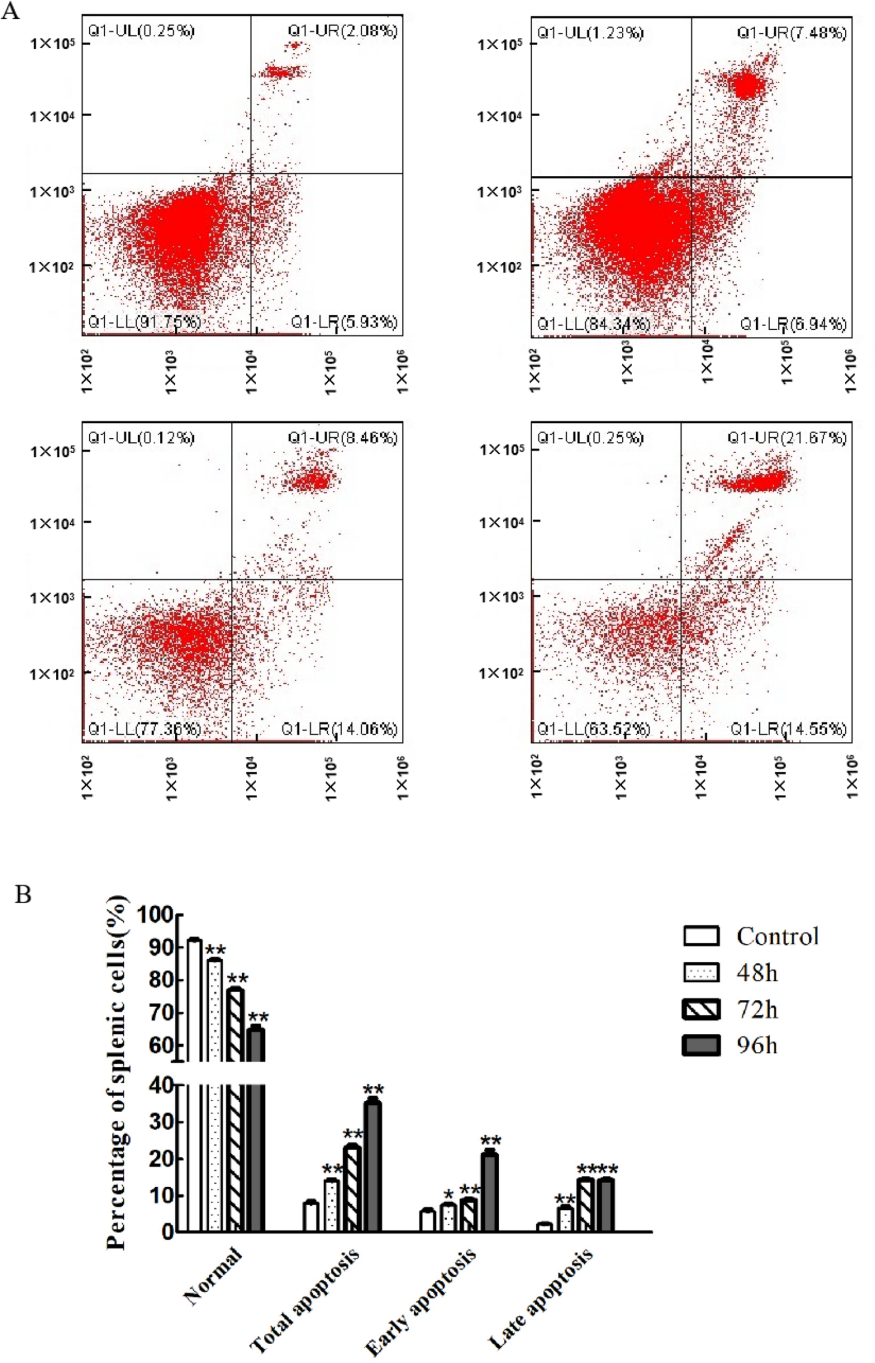
Effect of PRV on the percentage of splenocyte apoptosis. (**A**) The results of apoptosis detected by flow cytometry; (**B**) Apoptosis statistical analysis histogram.

It can be seen from Fig. 4 that, the proportion of early, lately and total apoptotic cells in the PRV infection group significantly increased by comparing with the control in the infection period. In the 48 hpi time point, apoptotic cells were significantly increased (*p*<0 05).However, the apoptotic cells extremely increased at the 72 and 96 time points(*p*<0 01). PRV could promote the production of splenocytes apoptosis.

### Relative mRNA expression levels related to apoptosis in the Spleen

**Figure 5:**
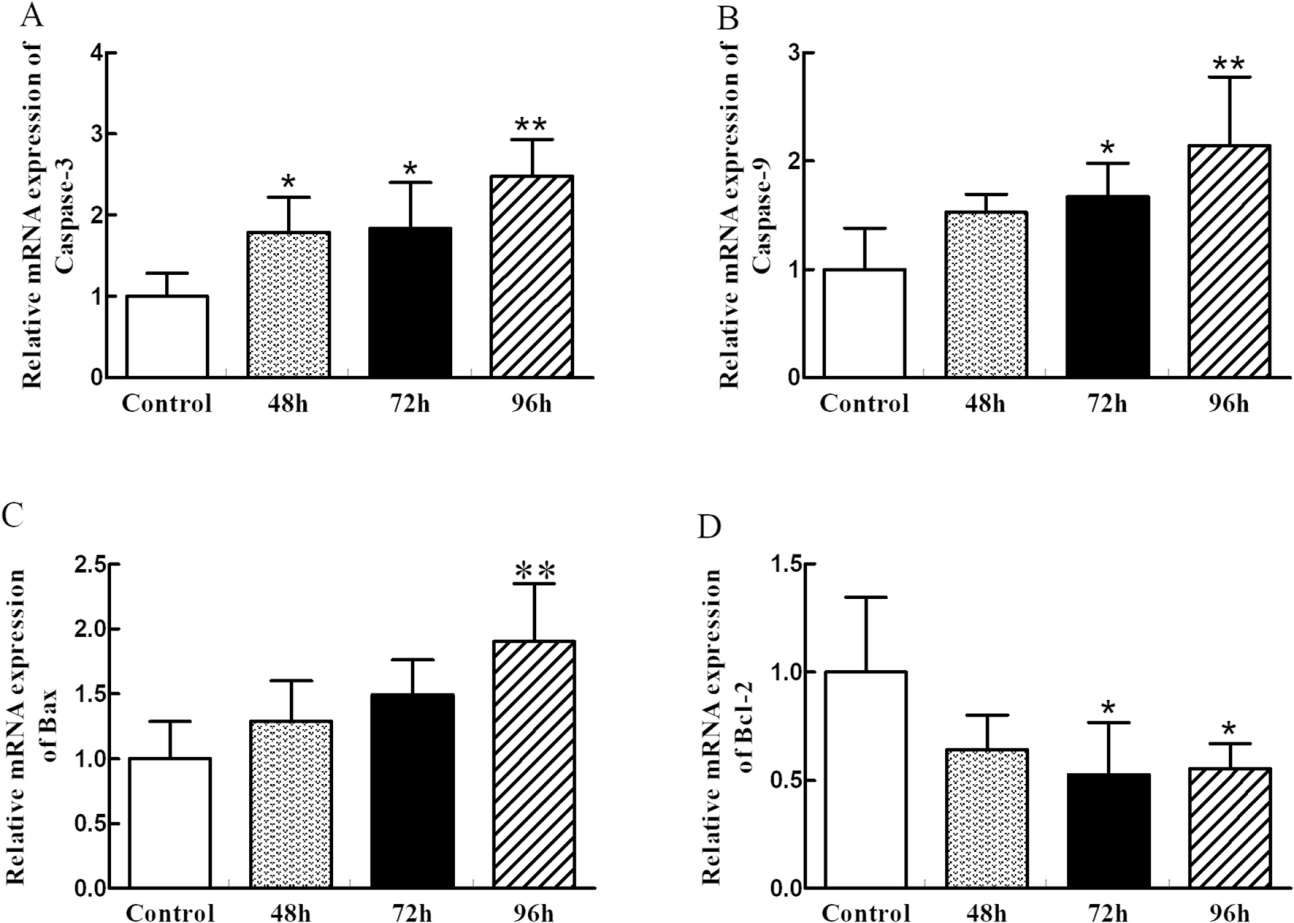
The relative mRNA expression of apoptosis-related factors caused by PRV.

From the results in Fig. 5, Caspase-3 relative mRNA expression levels in the PRV infection group at 48 hpi, 72 hpi and 96 hpi were all increased (*p *< 0.05 or *p *< 0.01) by compared with their respective controls. Furthermore, qRT-PCR results demonstrated that Caspase-9 mRNA expression levels in the PRV infection group were also up-regulated at 72 hpi and 96 hpi groups (*p *< 0.05 and *p *< 0.01) compared to that in the control group. Furthermore, the Bax expression levels of the PRV infection groups were not significant difference at the 48 hpi and 72 hpi time points compared to their respective controls (*p *> 0.05). However, we observed a significant increase in Bax levels at the 96 hpi time point for PRV infection compared with the gene in the control group. In addition, the mRNA expression of Bcl-2 in the PRV infection group decreased at the 72 hpi and 96 hpi time points compared to their respective in the control (*p *< 0.05 and *p *< 0.01).

### Effects of PRV on the proteins related to apoptosis in the Spleen

**Figure 6:**
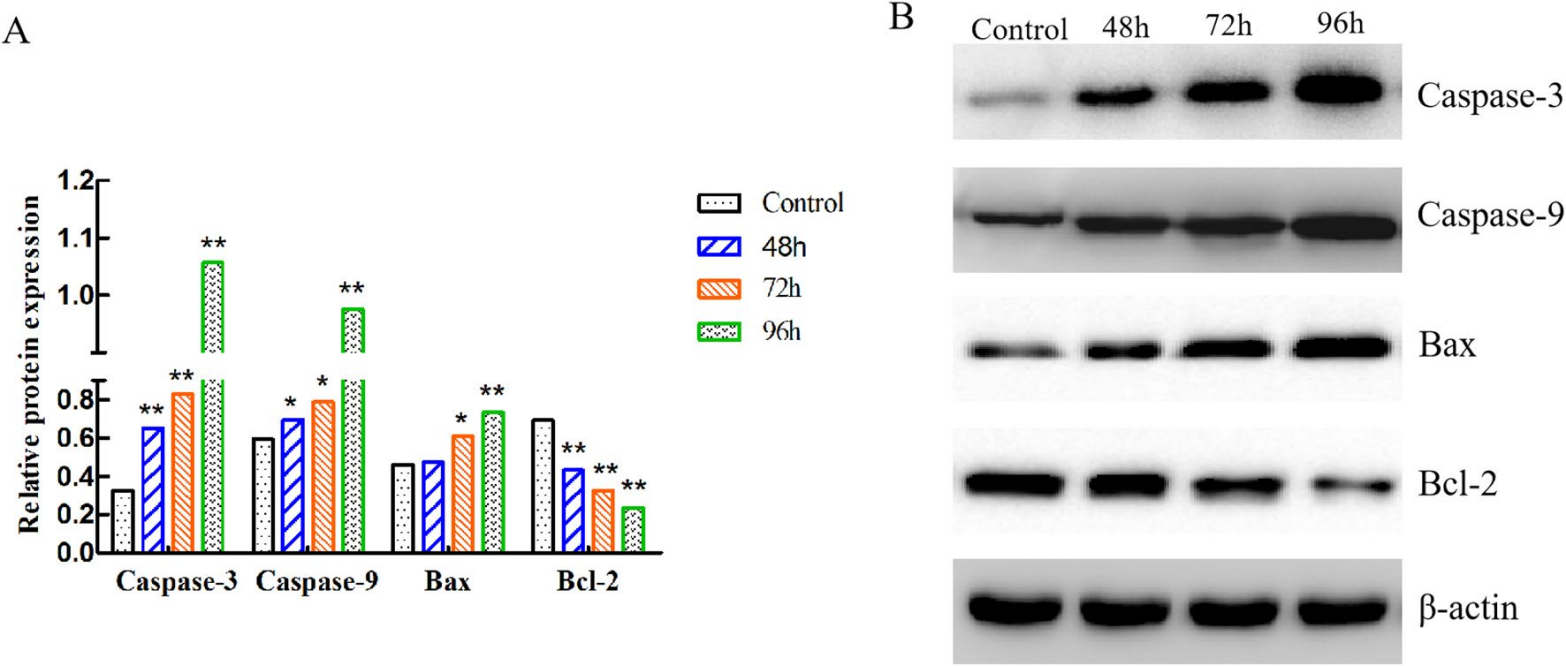
The expression levels of apoptosis-related factors induced by PRV in protein. (**A**) The relative protein amount of Caspase-3, Caspase-9, Bax, and Bcl-2 to β-actin. (**B**) Protein expression related to apoptosis detected by western blot.

According to the results in Fig. 6, Caspase-3 protein expression levels were significantly increased in the 48 hpi, 72 hpi, and 96 hpi PRV post-infection time points compared that in the control group. In addition, compared with their respective control groups, the PRV infection group showed a significant up-regulation of Caspase-9 at the 96 hpi post-infection time point. Moreover, the Bax expression in the PRV infection group remarkably increased at all the time points compared to their respective control groups. Furthermore, PRV down-regulated the protein expression of Bcl-2 in the 48 hpi, 72 hpi, and 96 hpi group by comparing them with the control groups.

## Discussion

The spleen is one of the major parenchymal organs affected by PRV. After PRV infection, the spleen mainly exhibits small white coagulative or lytic necrotic lesions^6^. In this study, we observed congestion and even inflammatory cell infiltration in the red pulp area of the spleen after PRV infection. This results was consistent with a study that showed PRV induces the tumor necrosis factor-α (TNF-α) mRNA levels and promoting of TNF-α secretion, which results in cell apoptosis^10^. The spleen is a substantial organ rich in mitochondria, an organelle that serves as the main source of ROS production. However, the mitochondria are also susceptible to ROS damage. ROS promotes the formation of lipid peroxidation, in which MDA is a key marker^4^. The oxidative stress induced by the out-of-balance between oxidants and antioxidants is an important feature driving the occurrence of a series of viral infectious diseases^5^. In our study after PRV was subcutaneously inoculated into mice, we observed that PRV promoted the production of ROS and MDA in the spleen of mice, reduce the production of non-enzymatic substances GSH, and at the same time down-regulated the gene expression of antioxidant enzymes (CAT, SOD, and GSH-Px), indicating that PRV can break the balance between splenocyte oxidation factors and antioxidant factors, hence induce oxidative stress in the spleen. Our finding was consistent with a study by Lai^3^ which reported that, ROS was generated in the PK15 cells infected with PRV and this caused DNA damage and cell apoptosis. Another study also reported that HSV-1, a member of the PRV genus, causes GSH depletion and increases ROS and lipid peroxidation levels^11^. It has been reported in both cellular and animal models that HSV-1 causes host damage through oxidative stress^12^.

High levels of ROS are the main cause of cell apoptosis under physiological and pathological conditions. PRV induces the oxidative stress and damage of DNA, thus affecting the expression of pro-apoptotic signaling molecules and key regulatory molecules of cell cycle^3^. Apoptosis is a mechanism for maintaining balance in vivo, and has a pivotal role in viral infection. Several viruses directly induce host cell apoptosis at the stage of infection, which may be a pathological factor related to infection. It is known that PRV infection triggered suicide or apoptosis of infected cells^13^. Generally, apoptosis is activated mainly in the late stage of infection by PRV. Moreover, studies have shown that PRV triggers ROS production and causing of apoptosis in autophagic-damaged cells, which indicated that ROS may be participated in the linking of autophagy and apoptosis in cells infected by PRV^14^. The results in this studies indicated that the PRV-HLJ strain caused oxidative stress in the spleen of mice, thereby inducing splenocyte apoptosis via ROS production method. Oxidative stress is associated with various cell death processes, including apoptosis, caused by viral infection. Hepatitis B virus (HBV) combines with the mitochondria through the C-terminal of X protein (HBX) to cause damage to mitochondrial DNA, and over-express the SIRT-1 gene for ROS production^15^. Interestingly, the Hepatitis C virus (HCV) causes damage to host cells in mitochondria by activating oxidative stress, and the damaged mitochondria are eliminated by autophagy^16^. In this work, our results indicated that the PRV-HLJ strain caused oxidative stress in the spleen of mice, thereby inducing splenocyte apoptosis. After infecting mice with the PRV-GXLB-2013 strain, they observed an increase in ROS production in the brain, spleen, and lung^17^. From these results, we speculated that there is a certain connection between the immune response caused by PRV and an organ being in a state of oxidative stress, however, the specific mechanism is not yet clear, and requires further research.

Virus infection activates Caspase-3, leading to cell apoptosis^18^. Caspases, a series of enzymes in the cysteine protease family, plays a pivotal function in the maintaining of homeostasis and regulating of programmed cell death in vivo. Caspase-3 and caspase-9 have been widely classified based on their known role in cell apoptosis^19^. Caspase-3 activation is essential for the promotion of cell apoptosis. Bax and Bcl-2 are two regulators in the process of releasing cytochrome C and mitochondrial integrity. Research has shown that excessive levels of ROS have a crucial role in activating the mitochondrial-dependent apoptosis signal pathway, which promoted the release of cytochrome C, reduces levels of mitochondrial membrane potential (MMP), damages cell respiratory function, disturbances the imbalance of energy metabolism, and finally apoptosis^20^. Apoptosis is inevitably related to the change of MMP. The decline of mitochondrial membrane potential is an important landmark event for apoptosis^21^. In this study we observed that after PRV injection the apoptotic markers (Bax caspase 3 and caspase 9) were increased. Similar results were obtained from the studies by Sun et al.^14^ which reported that PRV-induces apoptosis by increasing the apoptotic markers such Bax Caspase 3 and 9. 

The studies on the mechanism of PRV invasion are still unclear. The oxidative stress caused by the excessive generation of ROS in the spleen after being invaded by the virus is closely related to tissue damage. This was consistent with our experimental results which confirm that PRV infection caused spleen oxidative stress, thereby inducing splenocyte apoptosis. This study provides certain scientific data for further exploring the spleen damage caused by PRV and its pathogenic mechanism.

## Conclusion

Pseudorabies virus (PRV) is an immunosuppressive disease that causes significant damage to the pig industry as it leads to economic losses worldwide. Due its negative impact to the economy PRV has been of interest to virologists and neurobiologists who are concerned about PRV disease control especially in swine agriculture. However, for the scientific community to develop effective control strategies for PRV and its related disorders, knowledge on the PRVs pathogenicity is key. Therefore, our study investigated the effect of PRV on the spleen and found that Pseudorabies virus strain HLJ strain (MK080279.1) induces apoptosis in the spleen of mice via ROS production. This information will serve as the basis for the development of strategies to minimize the spread of the PRV or their harm to their hosts (Supplementary Information).

### Supplementary Information

Supplementary Information.

## Data Availability

Data will be made available upon reasonable request from the corresponding author.
