# Supplementary material for: Pseudorabies virus causes splenic injury via inducing oxidative stress and apoptosis related factors in mice
Source: Sci Rep. 2023 Dec 27;13:23011. doi: 10.1038/s41598-023-50431-7 (PMC10754911; doi:10.1038/s41598-023-50431-7)
Supplement: Supplementary file 1 — Supplementary Information. [file 41598_2023_50431_MOESM1_ESM.pdf]

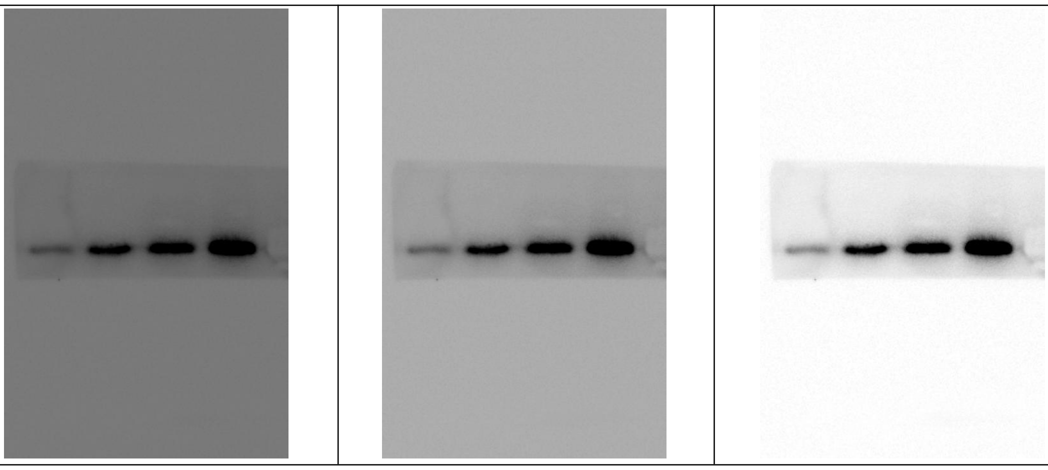

Caspase-3(Control, 48h,72h, 96h with different exposure time)

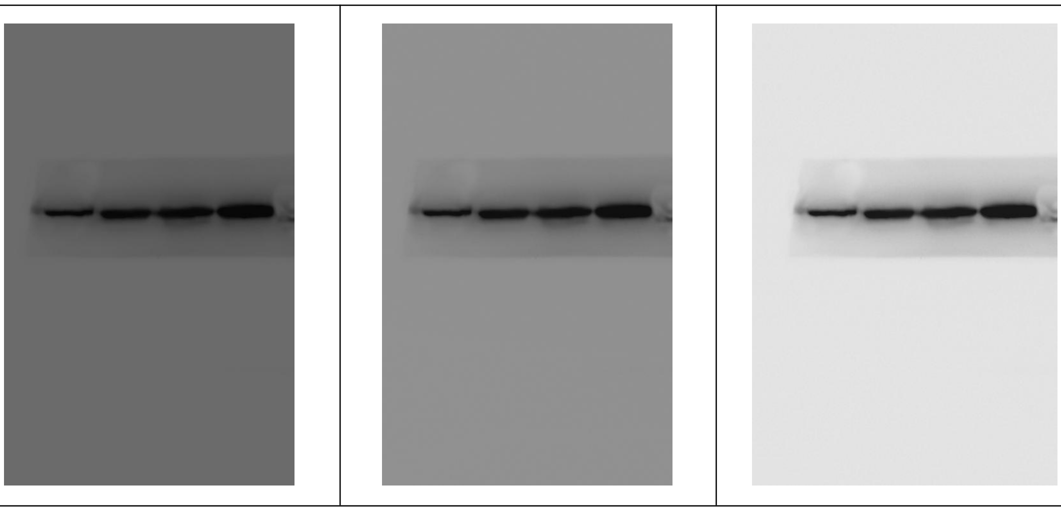

Caspase-9(Control, 48h,72h, 96h with different exposure time)

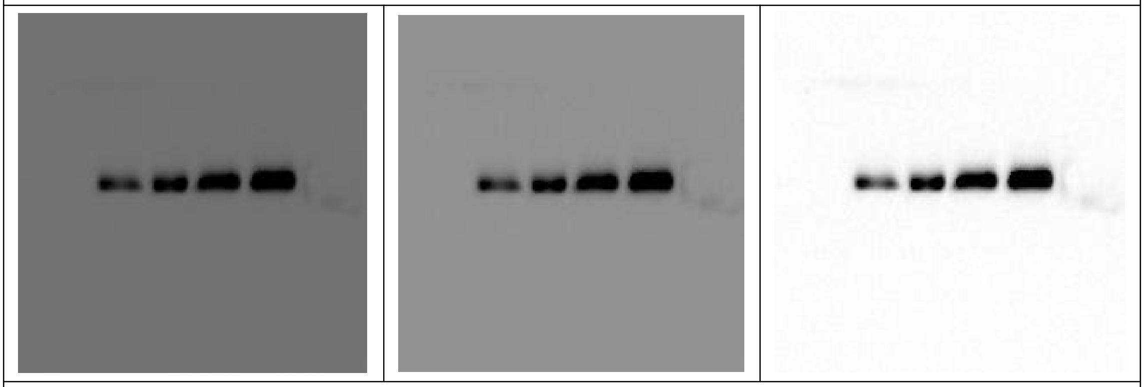

Bax(Control, 48h,72h, 96h with different exposure time)

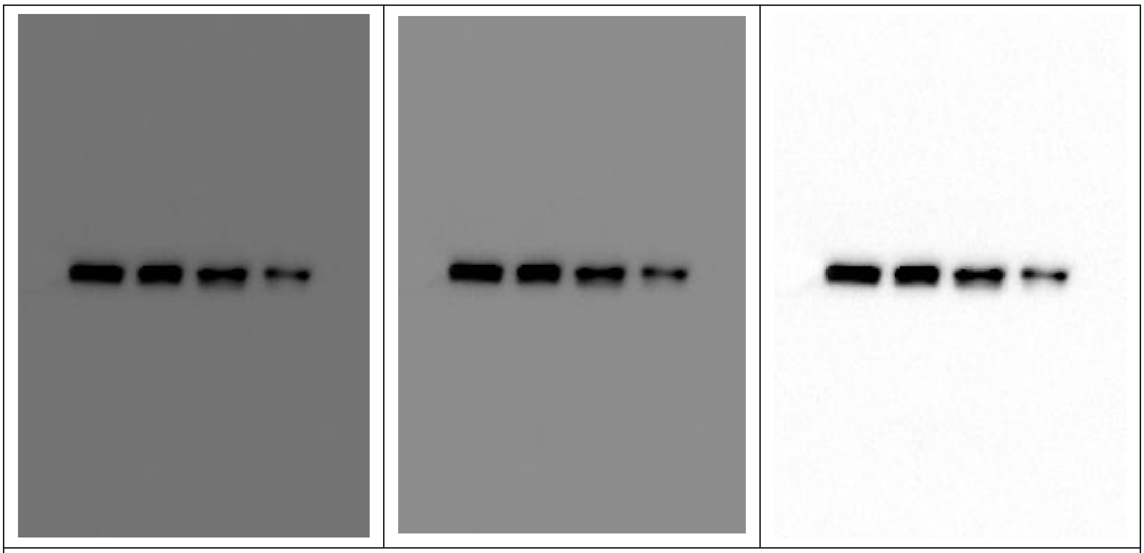

Bcl-2(Control, 48h,72h, 96h with different exposure time)

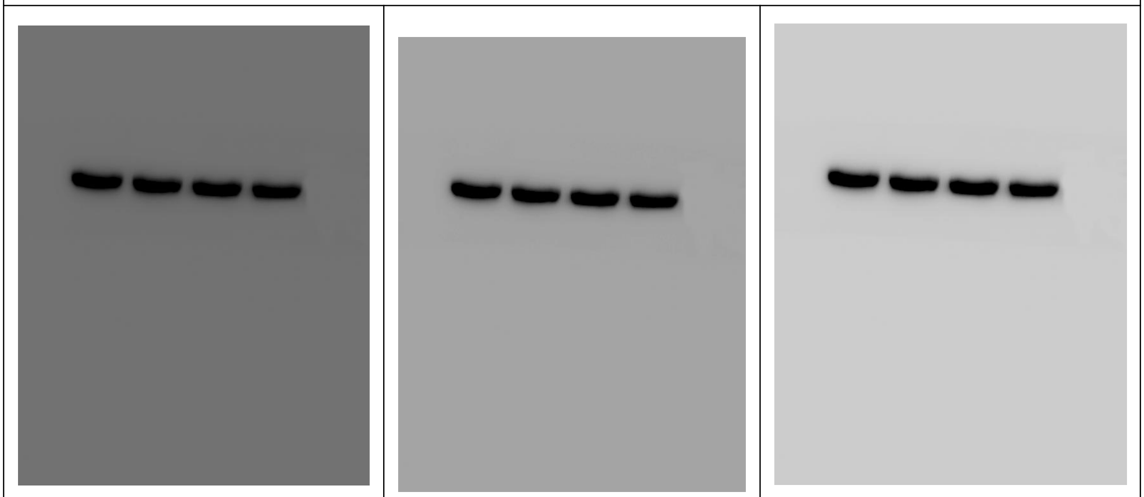

β-actin(Control, 48h,72h, 96h with different exposure time)
